# Clinical Algorithms and the Legacy of Race-Based Correction: Historical Errors, Contemporary Revisions and Equity-Oriented Methodologies for Epidemiologists

**DOI:** 10.2147/CLEP.S527000

**Published:** 2025-07-12

**Authors:** Laura J Horsfall, Paulina Bondaronek, Julia Ive, Shoba Poduval

**Affiliations:** 1 Institute of Health Informatics, University College London, London, UK

**Keywords:** clinical algorithms, race correction, health disparities, algorithmic fairness, algorithmic bias, lung function, spirometer, bias mitigation, bias detection

## Abstract

Clinical algorithms are widely used tools for predicting, diagnosing, and managing diseases. However, race correction in these algorithms has faced increasing scrutiny for potentially perpetuating health disparities and reinforcing harmful stereotypes. This narrative review synthesizes historical, clinical, and methodological literature to examine the origins and consequences of race correction in clinical algorithms. We focus primarily on developments in the United States and the United Kingdom, where many race-based algorithms originated. Drawing on interdisciplinary sources, we discuss the persistence of race-based adjustments, the implications of their removal, and emerging strategies for bias mitigation and fairness in algorithm development. The practice began in the mid-19th century with the spirometer, which measured lung capacity and was used to reinforce racial hierarchies by characterizing lower lung capacity for Black people. Despite critiques that these differences reflect environmental exposure rather than inherited traits, the belief in race-based biological differences in lung capacity and other physiological functions, including cardiac, renal, and obstetric processes, persists in contemporary clinical algorithms. Concerns about race correction compounding health inequities have led many medical organizations to re-evaluate their algorithms, with some removing race entirely. Transitioning to race-neutral equations in areas like pulmonary function testing and obstetrics has shown promise in enhancing fairness without compromising accuracy. However, the impact of these changes varies across clinical contexts, highlighting the need for careful bias identification and mitigation. Future efforts should focus on incorporating diverse data sources, capturing true social and biological health determinants, implementing bias detection and fairness strategies, ensuring transparent reporting, and engaging with diverse communities. Educating students and trainees on race as a sociopolitical construct is also important for raising awareness and achieving health equity. Moving forward, regular monitoring, evaluation, and refinement of approaches in real-world settings are needed for clinical algorithms serve all patients equitably and effectively.

## Introduction

Clinical algorithms are decision-making tools designed to assist health care providers in predicting, diagnosing, and managing diseases. These tools are widespread in health systems and can range from simple flowcharts to increasingly complex models deploying artificial intelligence (AI). One notable practice in the development of such algorithms is race correction, which adjusts clinical calculations based on the patient’s racial or ethnic background. In its simplest form, this involves applying a multiplicative factor to the calculated clinical value for individuals identified as belonging to a specific racial or ethnic group. These adjusted values influence critical clinical decisions, including diagnoses, treatments, and referral thresholds.^1^

However, race correction has come under increasing scrutiny for its potential to perpetuate health disparities and reinforce harmful stereotypes.^2^,^3^ For instance, race-based adjustments in estimated glomerular filtration rate (eGFR) increases the calculated value for Black patients by approximately 16% to match White counterparts. The biological conjecture was that the higher average serum creatinine levels, used to calculate eGFR, were due to greater muscle mass of Black Americans.^4^ This race correction artificially elevated kidney function in Black patients, which delayed disease diagnosis and eligibility for transplants.^5^ Critics argue that race correction generally lacks a robust scientific basis and is a sociopolitical construct based on physical characteristics, such as skin color and facial features, and an imprecise proxy for continuous genetic diversity and lifelong environmental exposures. Algorithmic race correction essentially overemphasis the role of biology often based on outdated and unproven theories. This overemphasis leads to a neglect of the role of true drivers of health disparities relevant to current health, such as socioeconomic factors and structural racism.^3^

In response, there is a growing momentum within the medical community to reassess and, where necessary, eliminate race-based adjustments in clinical algorithms. However, simply removing race-based adjustments may inadvertently worsen algorithmic fairness because the clinical goal and operational context of the model plays a significant role.^2^

This narrative review examines historical misconceptions, primarily from the United States and the United Kingdom, that have embedded race into clinical algorithms. We adopt an interdisciplinary analytic framework, drawing on historical epidemiology, public health ethics, and fairness metrics to examine the origins, consequences, and reform of race correction in clinical algorithms. We also incorporate recent guidance and highlight actionable strategies for clinical researchers, epidemiologists, and health data scientists to improve algorithmic fairness and equity in healthcare.

## Historical Background

Ironically, the lungs - organs highly influenced by environmental factors - were among the first to be mischaracterized by the flawed genetic determinism that underpins many race-based medical adjustments. The development of lung function measurement, as meticulously documented by the late Lundy Braun,^6–8^ illustrates how epidemiological oversights, such as confirmation bias, causal misinference and residual confounding, contributed to the emergence of race correction.

The origins of technical race correction in medicine can be traced to the mid-19th century with the invention of the spirometer by Dr. John Hutchinson at University College London.^6^,^8^ Hutchinson’s spirometer gained acclaim for quantifying the relationship between height and lung capacity through compelling visual data, lending both biological and statistical credibility to the device.

In the United States (US), the spirometer was quickly co-opted to reinforce racial hierarchies. Samuel Cartwright, a plantation physician, adapted the device to compare lung function between enslaved Black Americans and free White Americans. Using data that showed Black individuals had, on average, 20% lower lung function, Cartwright concluded that this disparity was evidence of Black people’s supposed biological fitness for enslavement.^9^ Subsequent researchers observed similar racial and ethnic disparities in lung function, but influenced by confirmation bias, attributed these differences to innate biology without critically examining alternative explanations.^6^,^8^ They overlooked residual confounding of their causal biological theory by social and environmental factors such as malnutrition, poor living conditions, and limited access to healthcare.

However, the nature above nurture hypothesis did not go unchallenged at the time. Black scholars, including prominent mathematician Kelly Miller, criticized these arguments. Miller dismantled the notion of a “malignant, capricious ‘race trait’” with mathematical precision, exposing the flawed reasoning underpinning these claims.^8^,^10^ However, these critiques did not receive the attention they deserved and the belief in race-based differences in lung capacity endured in the medical literature for the next two centuries.

By the 1960s, the rise of genetics as a dominant scientific paradigm further sidelined environmental explanations for racial disparities in health outcomes.^7^ A pivotal 1974 study published in *The International Journal of Epidemiology* compared lung function between Black and White asbestos workers but failed to account for the complex social and environmental factors at play.^11^ The study reported a 13% difference in lung function, which was subsequently encoded (with modifications) into spirometers and the corresponding software worldwide. These artificial uplifts in lung function for people identified as Black or Asian raised the bar for a clinical diagnosis and removed the role of true drivers of health disparities, such as socioeconomic factors and structural racism.^3^ Despite objections, such as those from Jonny Myer in apartheid South Africa, who highlighted social determinants over inherent racial traits, these adjustments have persisted and have potentially underdiagnosed lung disease in vulnerable minority populations across the world.^12^ Myer was an early advocate for universal lung function standards to ensure equitable disease prevention and worker compensation.

By the late 20th century, race-based adjustments were widely incorporated into clinical algorithms, influencing decisions ranging from kidney transplant eligibility to birth plans and cardiovascular treatments.^1^,^13^ However, the early 2020s marked a turning point in the scrutiny of race-based medical practice. Firstly, the COVID-19 pandemic disproportionately impacted minority communities in high-income countries. While early discussions centered on biological factors like vitamin D, attention soon shifted to the real drivers, such as intergenerational living, supported in part by the availability of large, representative health datasets.^14^ Then, the murder of George Floyd catalyzed widespread calls for systemic reform under the banner of the Black Lives Matter movement. These two events have brought global attention to racial inequities across healthcare, education, and economy. In response, medical institutions began reassessing their practices and initiating reforms in education, clinical training, and healthcare delivery to address racial bias. For the first time, clinical algorithms incorporating race have faced unprecedented levels of scrutiny, ushering in a critical reevaluation of their role in perpetuating health disparities.

## Modern Data Science and the Reproduction of Structural Bias

Despite growing awareness of racial bias in clinical algorithms, many contemporary data science practices continue to replicate discredited assumptions under the guise of neutrality. This has been powerfully critiqued by Ruha Benjamin (2019), who coined the term “The New Jim Code” to describe how seemingly progressive technologies can reinforce racial hierarchies.^15^ In the context of clinical algorithms, this manifests in models that exclude race as a variable but still encode racial bias through proxies, historical data, and structural inequities embedded in the healthcare system.

The illusion of neutrality in algorithmic design is sustained by the widespread belief that data are objective, apolitical, and detached from social context. But data are deeply shaped by the historical and institutional settings in which they are collected. Clinical datasets, for example, often encode longstanding disparities in healthcare access, diagnosis, and treatment. When these datasets are used to train algorithms without critical scrutiny, they risk reinforcing the very inequities they purport to mitigate. This dynamic aligns with Michel Foucault’s (1976) concept of biopolitics, where power operates through the regulation of populations and bodies by means of apparently neutral institutions, discourses, and technologies.^16^ Clinical algorithms, in this sense, are not simply tools for enhancing diagnostic precision; they are technologies of governance that can normalize structural racism under the banner of efficiency and scientific rationality.

For example, algorithms trained on electronic healthcare records (EHRs) may inherit biases from historical underdiagnosis or undertreatment of racialized groups. Even when race is excluded as an input, other variables, such as insurance status or comorbidity patterns, can serve as proxies, leading to disparate outcomes. Without deliberate efforts by researchers to identify and mitigate these patterns, algorithmic outputs may appear neutral while reproducing racial disparities in care.

This issue is particularly problematic in the case of proprietary algorithms and opaque AI systems, where the underlying data, model architecture, and decision-making are not publicly disclosed. Such opacity limits the ability of clinicians, patients, and regulators to scrutinize or challenge biased outcomes. It also undermines accountability, as developers can claim neutrality while concealing the assumptions and trade-offs embedded in their models. The lack of transparency in commercial AI tools applied to healthcare raises serious ethical concerns, especially when these tools influence high-stakes decisions such as diagnosis, treatment eligibility, or resource allocation.

## Clinical Context Matters

Amid growing political and societal pressure, numerous medical organizations in the US and Europe are actively reassessing their use of race in clinical algorithms, with some institutions and researchers initiating the removal of race-based adjustments entirely.^17–19^ Algorithms in fields such as obstetrics and nephrology quickly implemented race redaction by 2021 as the biological justification for racial adjustments was particularly tenuous.^13^ However, other groups have advocated for a cautious approach, warning that the removal of race - a strong predictive factor - could unintentionally exacerbate disparities.^20–23^

The transition to race-neutral Global Lung Function Initiative (GLI) equations demonstrates this delicate balance of aiming to improve equity while addressing concerns about the unintended consequences. In 2023, the European Respiratory Society (ERS) and American Thoracic Society (ATS) endorsed new GLI equations that entirely removed race as a variable by averaging lung function across groups.^24^ This transition from race-specific GLI-2012 to race-neutral GLI-Global equations is estimated to have enhanced health equity by improved recognition and early diagnosis of respiratory disease in Black people.^25–27^ A retrospective study that examined all patients listed for lung transplantation in the US between 2009–2015 found evidence that race-neutral equations may also improve access to lung transplantation in Black patients.^28^ In terms of lung-transplant priority, race-neutral equations were predicted to reduce inequities by reducing wait times for Asian and Black candidates by 4.3 days while increasing times for Hispanic and White candidates by 1.1 days.^26^ Eligibility for payments for impairment-based compensation were also estimated to double in Black adults and decrease by 25% in White adults.^26^

Despite these potential equity improvements, concerns regarding the unintended consequences of race-neutral GLI equations persist. Potential issues include overdiagnosis, exclusion of minorities from professions requiring lung function tests, denial of certain treatments such as lung cancer resection, and increased life insurance premiums.^29^ For example, modeling studies have indicated that race-neutral equations may increase disqualifications from professions requiring lung function tests, such as firefighting for Black adults while decreasing disqualifications for White adults.^26^ Another study presented hypothetical scenarios to American surgeons and found that switching to race-neutral PFT may reduce lung cancer surgery referrals for Black patients from 79% to 53%.^22^

Following a comprehensive review of the literature on race and ethnicity in clinical algorithms, The Agency for Healthcare Research and Quality (AHRQ) in the US provided a balanced perspective in 2024, concluding that the benefits and risks of race removal depend heavily on the specific clinical context.^2^ This suggests that, while race-based algorithms can perpetuate health disparities, their complete removal may inadvertently lead to underdiagnosis or undertreatment in certain populations. With growing confusion regarding how to approach race and ethnicity in clinical algorithms, a panel of 14 leading experts and stakeholders convened to provide some clarity.^23^ The outcome is a living document: The Guidance for Unbiased predictive Information for healthcare Decision-making and Equity (GUIDE).^23^ The guidance is intended as a framework to consider trade-offs in the inclusion or exclusion of race in clinical algorithms and distinguishes algorithmic bias from fairness. The guidance cautions against race redaction without first exploring the context and goal-specific impact on algorithmic bias and fairness.

Therefore, researchers are tasked with carefully examining algorithmic bias on a case-by-case basis, considering the full pipeline from conception to dissemination.

## How Can Epidemiologists Contribute to Improving the Algorithmic Fairness?

**Figure 1:**
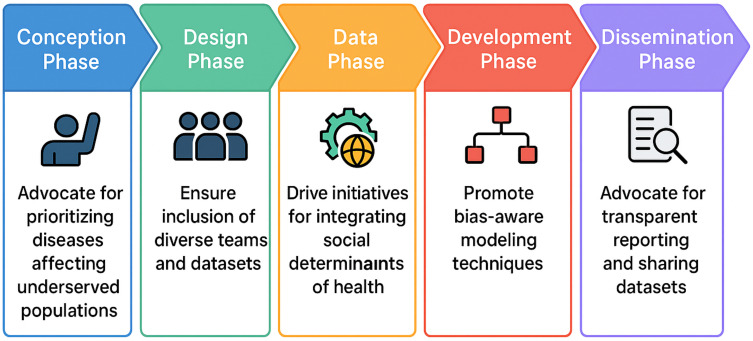
An adapted framework highlighting the roles and responsibilities of epidemiologists and health data scientists in improving algorithmic fairness.

In 2019, the FDA mapped the Total Product Lifecycle (TPLC) approach on the US regulation of medical devices onto clinical algorithms using AI that could be considered devices.^30^ The comprehensive pipeline has since been updated to incorporate equity and methods to reduce bias in every phase.^30^ This ranges from considering which diseases to target to post-market performance surveillance. We adapted the framework and highlighted the roles and responsibilities of epidemiologists and health data scientists in this process. A summary is provided in Figure 1.

### Conception

During the conceptualization of clinical algorithms, developers should consider health conditions and relevant care processes and prioritize diseases that disproportionately affect underserved populations. Engaging patients and community stakeholders from historically marginalized racial and ethnic groups during this initial phase could help identify disease-specific inequities and ensure that the algorithm addresses the unique challenges faced by these communities.^31–33^ Beyond engagement, involving co-production processes which entails involving patients and representatives from underserved populations directly and throughout the development processes to increase both transparency and inclusiveness of the algorithms. Co-production means that any intervention (here algorithm) is informed by lived experiences and context-specific knowledge that may not be captured by medical data alone. The goal of the algorithm also needs to be clarified at an early stage to understand potential issues around bias versus fairness, as highlighted in The GUIDE.^23^ In the prediction context, bias refers to variations in accuracy across subgroups, due to a lack of validation of model predictors in minority groups. Whereas unfairness may arise in limited resource situations, where there are conflicts between fairness and priorities. For models where the primary goal is shared patient-clinician decision-making (eg, prostate biopsy referral), the main concern is bias due to differential accuracy across subgroups, resulting in low specificity of prediction model outcomes and over-investigation of individuals from minority groups.^34–36^ Whereas both bias and unfairness can arise in models intended to allocate finite health resources (eg, transplant prioritization). High accuracy in this context may not necessarily lead to equitable distribution of resources.^37^

### Design

#### Diverse Teams and Samples

Once the condition and goal have been clarified and the next phase considers how the algorithm is designed. Involving patients and community members from historically marginalized racial and ethnic groups in initial qualitative research or algorithm co-development is central to creating inclusive and equitable healthcare solutions.^31^,^32^ For example, qualitative research suggests that patient-led approaches during the design phase may have avoided the incorporation of race in vaginal birth after c-section (VBAC) calculators that led to racial inequities in counseling.^38^ Researchers and developers who engage and recruit diverse teams of professionals and public members early in the process can draw on a wide range of perspectives, experiences, and cultural insights to proactively identify input variables, biases, and gaps in algorithm design and evaluation.^39^,^40^ This ensures that the algorithms are culturally appropriate, accessible, and effective for diverse communities.^39–41^ Engaging diverse communities in algorithm development will also require work to improve public trust in health research, data collection and use of health data in clinical algorithms, which historically have been lacking due to unethical research and neglect of diverse groups in recruitment to clinical research.^42^,^43^

New clinical algorithms are increasingly expected to demonstrate effectiveness, equity, and value for money in the real-world setting, which requires core expertise beyond epidemiology, medicine, and health data science during the design phases. For example, in their comprehensive review of the evidence base, the AHRQ emphasized the need for a move away from quantitative simulation and more direct observation and evaluation of the downstream consequences of clinical algorithms.^2^ By broadening the scope of expertise involved in the algorithm design-phase to include expertise in clinical practice, qualitative research, health equity, health economics, trials, and implementation science, researchers can better anticipate and address potential challenges in clinical application and patient outcomes.

It is also important to identify or design the collection of a large representative dataset with minimal potential for current and historical bias. Ensuring the data represents the full diversity of the underlying population with large samples for minority groups is particularly important for accuracy and downstream mitigation. For example, in 2018, the AMA revised the pooled cohort equations for CHD by using a more diverse dataset.^44^ Although the original algorithm overestimated the risk for most patients, potentially leading to unnecessary treatment, the new version was significantly better at predicting risk, especially for Black Americans. A subsequent investigation reported that the revised algorithm improved equity by reducing differences in risk assessment and recommendations for statin use between Black and White patients.^45^

One of the advantages of using routinely anonymized EHRs for algorithm design in more socialized healthcare systems with fewer access restrictions, such as the UK, is that it provides researchers with access to large longitudinal datasets that are broadly representative of the underlying population. Many of the current QRISK algorithms implemented by the UK’s National Health Service for cancer, cardiovascular and kidney disease were derived using representative EHRs from primary and secondary health care settings.^46^,^47^ There have however been concerns about QRISK algorithms generalizability due to variation between general practices in coding style, completeness of data and patient case-mix (variation in risk factors for disease).^48^ Given the uncertainty, research has led to suggestions that these tools should be used with additional input including clinical interpretation and incorporation of causal risk factors that better capture the unmeasured heterogeneity between different general practices.^48^

Using data from the intended settings of an algorithm is important. If an algorithm is developed using data from populations with regular healthcare access but deployed in underserved communities with barriers to access including language barriers and discrimination,^49^ it risks perpetuating inequities due to differential access and disease detection. The age of the data also matters, as historical data often contain biases from misclassification, missing data, or historical inequities in care access and quality.^50^ Additionally, we should consider whether label bias affects the outcome, where measured and true outcomes may systematically differ by race or ethnicity.^23^ Such inaccuracies can lead to biased algorithms that misrepresent disease prevalence or severity across groups.

#### Measuring Race and Ethnicity

When developing clinical algorithms, it is important to consider the justification for incorporating race or ethnicity and offer a clear explanation when publishing findings. Specifically, consider whether including these factors might reinforce the flawed concept of race as a biological construct or if it was necessary to represent the impacts of systemic racism to address inequalities.^2^

If there are strong reasons to include or explore race for bias detection and mitigation, the next consideration is the measurement. Epidemiologists and health data scientists involved in algorithm development frequently work with pre-collected data using broad ethnic and racial categorizations and therefore have limited influence over how race and ethnicity were originally recorded. With routinely collected EHRs, where the data are intended for patient management rather than research, and data collection methods vary between patient- or clinician-collected, developers often deal with misclassification and missing data, which can hinder the evaluation of algorithmic bias and fairness across groups. While difficult to address in the short-term, it is possible to advocate for better explanation to the public as to why accurate ethnicity data needs to be collected, consistent collection and improved quality of ethnicity data as members of the HDRUK are doing in the UK.^51^ In countries such as France, Canada, and the Netherlands, the collection of data on protected characteristics such as race and ethnicity is often prohibited; therefore, addressing bias in these settings is not possible.

### Development

#### Adding or Replacing Variables

In some contexts, removing race from an algorithm may have no major impact on the model accuracy. A prospective cohort study of White, Black, Hispanic, and Asian adults found that race-neutral equations had similar accuracy to the race-specific version in terms of predicting chronic lower respiratory disease events and mortality.^52^ Initially developed in 2007, the VBAC calculator included race as a variable, assigning Black and Hispanic women a 15–18% lower likelihood of success than White women, despite no clear biological basis for this adjustment.^13^,^53^ Further external validation of the calculator in ethnically diverse cohorts in the US, Canada, and Sweden found no evidence that adding race categories meaningfully improved model performance.^54^,^55^

Several studies have shown that replacing race with other clinical variables can improve performance in terms of accuracy. For example, the race-neutral VBAC calculator introduced in 2021 replaced race with clinical factors such as chronic hypertension while maintaining equivalent model performance.^56^ In 2020, the American Heart Association (AHA) introduced the PREVENT model, a race-neutral alternative developed using over six million diverse electronic health records.^57^ By replacing race with biomarkers, medication history, and metrics of social deprivation, PREVENT demonstrated predictive performance comparable to that of the earlier ASCVD model across racial groups. However, improved or maintained model performance may not always translate into algorithmic fairness. For instance, while transitioning from ASCVD to race-neutral PREVENT maintained accuracy, it halved high-risk estimates for Black adults (10.9% vs 5.1%), potentially limiting their access to statins and other preventive interventions.^58^ Given the well-documented barriers Black patients face in accessing healthcare, some cardiologists have raised concerns that PREVENT could inadvertently worsen treatment disparities and exacerbate health inequities.

Accurately understanding and measuring causal social and biological determinants in large datasets is essential for developing clinical algorithms that reflect true health disparities, ensure equitable care, and avoid perpetuating biases or inaccuracies in decision making. The challenge for algorithm developers is identifying and measuring these variables and will likely require involvement of underserved communities in design as discussed above.

#### Social Determinants

Using race as a proxy for genetic or biological factors oversimplifies the complex social determinants of health (SDoH), which are often the true causes of observed differences in health outcomes.^59^ For example, a study that included 48,170 NHANES participants found that adjusting for several SDoHs, including income, food security, and education, completely mediated the 60% higher premature mortality observed for Black people relative to White.^60^ However, social determinants are not reliably coded in large-scale EHRs that are increasingly used to develop and validate clinical algorithms.^61^,^62^ While measures of social deprivation can be inferred from address data, these are often outdated, derived for a given area (not household), and sometimes categorized into quantiles, further aggravating precision. For example, the largest providers of UK EHRs include a composite measure of social deprivation determined at the area level, divided into quantile scores and last updated in 2011 following the Census.^60^,^63^ This is the deprivation measure used in the most recent QRISK CVD tool updated in 2024.^46^ However, the PREVENT score demonstrated that even an imprecise measure of SDoH can replace race and improve or at least maintain accuracy, although there are still disagreements regarding the outcomes for CVD treatment equity.^58^

Researchers have applied large language models (LLMs) to extract SDoH from EHR codes and free-text data with some success.^64^,^65^ For example, a machine learning (ML) analytic pipeline was applied to the EHRs of 10,192 US-based patients to estimate an individualized polysocial risk score (iPsRS) and help identify type II diabetic patients at a high social risk for hospitalization.^65^ The team applied causal AI techniques to identify modifiable risk factors and optimize algorithmic fairness. They found that individual-level SDoH (eg, marital status, financial, and educational status) was more important than contextual measures (eg, murder rate per 1000 population) for predicting hospitalization, but these had high levels of missingness in the EHRs. For instance, housing stability had a particularly strong predictive value but was missing in 58% of the cohort.

Rather than relying on routine clinical data to derive SDoH, external linkages with existing environmental, educational, and social data sources at the household or person level are arguably the most effective and unbiased way to obtain a more complete picture. Linking external data with electronic EHRs faces challenges in terms of accuracy because of inconsistent and incomplete data and acceptability, as patients and providers may have concerns about privacy and data use and governance. A lack of standardized protocols for data integration can exacerbate these issues. Future research on data linkage should focus on developing robust data standards and interoperability frameworks to enhance data accuracy, governance and public trust.^66–68^ Additionally, increasing stakeholder engagement and education can improve the acceptability and support of integrating SDoH data into EHRs.

Supporting the integration of external data sources, deployment of AI techniques, and the promotion of non-clinical data recording by healthcare professionals will all enhance capacity to capture SoDH and produce more precise, fair algorithms.

#### Biomarkers and Omics

In addition to better measuring the external environment, many researchers advocate the inclusion of biomarkers, including genomics, to create race-neutral algorithms by focusing on individual genetic profiles rather than ethnic and racial categories. These algorithms can theoretically reduce health disparities by identifying genetic risk factors specific to individuals rather than relying on racial generalizations that are a poor proxy for continuous human genetic diversity.

However, early examples of this personalized approach were not encouraging in terms of accuracy. For example, one of the first genetic tests approved in the US for the purpose of guiding treatment, in this case irinotecan chemotherapy, produced a “null” result for 20–30% of people with recent African ancestry compared with 0% for White Europeans.^69^ Subsequent investigations on replacing or supplementing race with genetic data have shown more promise with improved kidney graft failure prediction^70^ and warfarin dosing for Black patients.^71^

Genetics plays a strong and relatively simple role in the outcomes of these three examples. For more complex diseases, such as CVD, the explanatory role of genetics is often weaker and multifactorial, involving many genetic variants generally compiled by researchers into disease-specific polygenic risk scores (PGS). The addition of PGS to the original CVD ASCVD risk tool resulted in modest improvements in 10-year CVD prediction for all ethnic categories examined (net risk reclassifications of 3–9%), but a limited impact on reducing ethnic variation in the C statistic used to measure model discrimination.^72^ In general, the portability of these tools, typically trained in European populations, to other racially and ethnically defined groups is demonstrably low.^73^ A recent analysis of the general PGS landscape has highlighted the poor performance of PGS in screening, prediction, and risk stratification.^74^ The cost-effectiveness of integrating genomic screening with clinical algorithms remains a subject of debate, alongside the issue of the equitable distribution and uptake of these services across diverse healthcare systems. By focusing on PGS and not questioning what we are attempting to measure, there is also the risk of reinforcing the genetic determinism that underlies problematic race-based algorithms. On balance, while genetic and PGS approaches may hold value in developing race-neutral algorithms for health outcomes with a strong genetic component, the value for more complex multifactorial diseases seems less promising. Focusing on methods to identify and incorporate strong SDoH may hold more value for developers of complex disease/prognosis algorithms.

The incorporation of circulating biomarkers has also been studied as an alternative to race in clinical algorithms, particularly in the field of kidney function. Following evidence of harm in terms of timely referrals, exclusion from trials, and kidney transplant lists, several major bodies, including the National Kidney Foundation, American Society of Nephrology, and the UK National Institute for Health and Clinical Excellence, simultaneously recommended the redaction of race from clinical algorithms estimating eGFR.^4^,^75^ Studies have characterized several biomarkers that may offer a more accurate and inclusive approach to estimating kidney function and other health metrics without relying on race as a variable.^76^,^77^ Cystatin C, beta-2 microglobulin, and beta-trace protein are emerging as promising alternatives for developing race-neutral algorithms for estimated glomerular filtration rate (eGFR).^76–78^ For example, race-neutral eGFR equations with creatinine and cystatin C are more accurate and result in smaller differences in observed versus eGFR between Black and non-Black patients than equations without race with either creatinine or cystatin C alone.^78^ A systematic review on bias and accuracy in US-based eGFR equations supported the role of cystatin C but emphasized that the solution to the disproportionate burden of kidney failure on Black Americans is multifaceted and should also include effective disease prevention, tackling SDoH, and systemic racism.^79^

Before incorporating new biomarkers, it is important to consider the evidence that these measurements are racially invariant to minimize the risk of bias in the algorithm output.^80^ Furthermore, any improvements in accuracy and reduction in bias resulting from the addition of biomarkers to existing algorithms will need to demonstrate value for health systems with finite resources. Even inexpensive biomarkers that significantly improve model discrimination may not be cost-effective.^81^

### Bias Detection

An initial examination of the training data can identify imbalances or underrepresentation of certain groups. Analysts can identify potential algorithmic bias by breaking down model performance metrics (eg, C statistic, AUC) and explanatory power (eg, R^2^, pseudo R^2^) by ethnic and racial categories. For example, the most recent iteration of a CHD risk tool used extensively across the UKs National Health Service reported the C statistic for ethnic groups.^46^ This highlighted clear differences in model performance, and the addition of nine additional clinical variables had no strong impact on reducing these differences.

In addition to the performance measures, the clinical context and goal of the algorithm could necessitate multiple bias metrics.^82^ These might include anti-classification (effect of protected attributes on predictions), calibration (difference between predicted risk and observed risk), optimizing equalized odds (sensitivity and specificity are similar across all groups) and predictive parity (positive predictive values are similar across groups). However, the choice requires careful consideration of the goal of the model owing to compatibility issues.^82^ GUIDE recommends against the use of classification measures that are sensitive to disease prevalence (eg, sensitivity and specificity) when exploring bias.^23^ Model inputs may also have undergone previous race adjustments, such as lung function tests. It is important to identify such variables and evaluate the possibility of eliminating adjustments in the main model, or as part of a sensitivity analysis.

### Statistical Mitigation

For researchers that identify potential issues with algorithmic bias and fairness,^23^ a rapidly increasing number of statistical mitigation strategies have been reported to improve fairness for author-selected metrics.^83^,^84^ In some contexts, these algorithmic debiasing methods produce fairer models than removing race from training data.^85^

Opportunities for statistical bias mitigation broadly fall into three stages for AI (and traditional) modelling strategies: preprocessing, in-processing and postprocessing (Table 1).^83^,^86^

**Table 1:** Comparison of Data-Based Bias Mitigation Techniques Used in Clinical Algorithms Development

Development Stage	Description	Reported Effects or Trade-offs
Preprocessing	General data cleaning and conversion of raw data into a format appropriate for analysis. Techniques include probability weighting, oversampling, and SMOTE.	Effective in addressing representativeness issues but may introduce synthetic data artifacts.
In-processing	Techniques applied during the training phase to reduce bias and introduce fairness goals. Methods include separate models by race, recalibration, and adversarial debiasing.	Can harmonize error rates but may lead to overfitting or miscalibration.
Post-processing	Adjusting outcomes in a group-dependent manner after training without altering the classifier or data. Methods include race-specific thresholds and recalibration.	Partially reduces inequities but often insufficient alone.

**Note**: Data from these studies.^83^,^86^

#### Preprocessing

Preprocessing refers to general data cleaning and the conversion of raw data into a format appropriate for analysis. It is possible to address problems identified with representativeness at this stage by rebalancing the data. For example, probability weighting or oversampling underrepresented groups and under sampling over representative groups. An alternative approach, termed the Synthetic Minority Oversampling Technique (SMOTE), generates plausible synthetic copies of the minority group through interpolation, maintaining diversity and not simply duplicating samples.^87^ The random forest ensemble approach rebalances data by taking bootstrap samples from the minority group, followed by equal samples from the majority.^88^ All these techniques perform well in the presence of marginal bias based on the results of simulations and application to real health data.^88^

#### In-Processing

In-processing refers to the techniques applied during the training phase of a model to reduce bias and to introduce fairness goals. The simplest option is to develop models separately by race or ethnicity, but this assumes that race necessarily interacts with other variables and can lead to implausible predictions owing to overfitting, particularly for smaller minority samples.^44^ Another option is to recalibrate the model to assign a greater weight to underrepresented groups.^89^ Researchers found that this method improved group-level calibration but also increased error rates when deriving new ASCVD risk estimators in the context of guideline-recommended thresholds.^89^ Forcing the use of racially invariant priors instead of fully deriving the algorithm from the training data can help reduce bias in AI models.^30^ This approach was adopted in the development phase of the first FDA-authorized autonomous AI diagnostic system aimed at the early detection of diabetic retinopathy in the primary care setting.^80^ The equalized odds constraint method selects a model that minimizes group-level differences in the true positive/negative and false positive/negative rates. This method successfully harmonized error rates for guideline-recommended thresholds in a 10-year ASCVD risk prediction model, but at the expense of increased miscalibration.^89^ A Quasi-Pareto Improvement approach was proposed to enhance the subgroup prediction performance and fairness of algorithms trained using thyroid nodule ultrasound image dataset.^90^ Adversarial debiasing in an ML method applies penalties to model iterations, where the output can predict sensitive characteristics, such as race.^91^ This method performed well in ethnically debiasing COVID-19 predictions relative to other benchmarks using complex EHR data, achieving the best results with respect to equalized odds.^91^

However, in processing mitigation may not be sufficient to prevent bias in isolation. For instance, to address the issue of implausible CVD scores in Black Americans from an earlier algorithm, one group identified newer, more diverse cohorts and applied elastic net regularisation to select predictors and accommodated non-proportional hazards to reduce overfitting.^44^ The authors reported that these adjustments improved calibration and plausible risk scores across subgroups, but the statistical measures were not sufficient without new diverse cohorts.^44^ In another example, adding a fairness constraint to penalize predictions that rely on race-reduced disparities in a model for postnatal depression were outperformed by pre-processing methods.^85^

#### Post-Processing

Most postprocessing techniques only adjust the outcomes in a group-dependent manner after training without altering the classifier or data. However, these post hoc mitigations are usually insufficient. For example, after identifying ethnic biases in a previously validated ML opioid misuse classifier, the research team attempted two post hoc mitigation experiments to reduce disparities in the false-negative rate.^92^ First, they applied race-specific thresholds and then recalibrated them according to racial subgroups. These methods only partially reduced inequities in treatment and education referrals and emphasized the need to consider bias across the full development pathway. Other methods, that are beyond the scope of this review but worth highlighting, include self-correcting LLMs.^93^

### Dissemination

#### Reporting

To ensure fairness and accountability in clinical algorithm development, there needs to be strong emphasis on transparent and inclusive reporting. Sharing code and de-identified datasets fosters transparency, enabling independent validation, bias detection, and reproducibility. The lack of transparency, particularly for proprietary AI, was highlighted by the AHRQ in its recent review as a barrier to patient understanding and trust, implementation and bias evaluation.^2^ The use of precise and inclusive terminology can also help address historical injustices and systemic inequities in health outcomes. A systematic review of articles published in *The Epidemiology* and *American Journal of Epidemiology* between 2020 and 2021 aimed to understand how race, ethnicity, and similar social constructs were operationalized, used, and reported.^94^ Although this review focused on the treatment of race as a confounder in causal research, there are several relevant recommendations on reporting that we have adapted and expanded for algorithm dissemination, as follows:

##### Clearly Define the Model’s Goal and Data Collection Methods

Specify whether the model is intended for shared decision-making or resource allocation. Detail the methodology used to collect racial data, such as self-identification, observation, survey items, or clinical assignment, and where feasible, adopt standardized classifications like those used in censuses.

##### Report Health Outcomes by Racial Categories Transparently

Present health outcomes for all racial categories alphabetically to facilitate comparisons of disparities without false hierarchies. Avoid aggregating racial groups unless there is compelling justification, particularly when differences exist in the prevalence or incidence of health outcomes. This practice ensures transparency and enhances the predictive accuracy of the model. If racial categories are combined, provide a clear rationale, such as addressing model nonconvergence, and use descriptive terms for the merged groups instead of labeling them as “other.”

##### Evaluate and Report Model Performance by Racial Groups

Provide detailed metrics of model performance (eg, C-statistic, AUC, calibration slope/intercept) and explanatory power (eg, R²) by race and ethnicity. Explain how the bias was identified and mitigated. Consider tools like PROBAST to assess the risk of bias and applicability systematically.^95^

##### Ensure Transparency Through Open Sharing

Improve transparency by sharing code and de-identified datasets with permanent identifiers to allow for independent validation, bias assessment, and reproducibility. Publish these resources with safeguards such as anonymization and governance frameworks to maintain privacy and foster ethical and secure collaboration.

##### Address Historical Context in Reporting Disparities

When discussing racial and ethnic disparities, acknowledge historical injustices, as recommended by the American Medical Association (AMA).^96^ Use appropriate terminology, such as racism, structural racism, racial equity, or inequity. Refer to guidelines such as those provided by the AMA and APA for inclusive and accurate scientific language when referencing race and ethnicity.^96^,^97^

#### Education

One of the most powerful tools available to those of us involved with training the next generation of epidemiologists and health data scientists is education. Educational interventions on race have shown significant potential in reducing biological essentialism among university students, which can lead to racial bias in health care. For example, US undergraduate students taught to perceive race as a social construct rather than a biological characteristic, exhibited greater emotional distress over social inequality, and were more motivated to address these issues.^98^ Conversely, students primed to view race as a biological construct were more likely to see inequalities as unproblematic.^98^ Evidence from a study that randomly assigned undergraduate students to an educational intervention on race also supports the role of education in reducing biological essentialism.^99^ Other studies have focused on the impact of genetics education on high-school students. They found that traditional genetics education, which often emphasizes genetic differences between races, can inadvertently reinforce biological essentialism.^100^,^101^ However, when the curriculum was adjusted to highlight the social and environmental factors that influence genetic traits, students were less likely to adopt essentialist views. This suggests that the way genetics is taught can significantly influence students’ perceptions of race.

Institutions delivering education in epidemiology and health data science can leverage existing evidence to enhance undergraduate and postgraduate courses by incorporating comprehensive education on race as a sociopolitical construct. Educational frameworks should also include sessions on equity, algorithmic bias, and fairness-aware design. This approach can help students understand how biases, stemming from historical inequities or biased data, manifest in algorithms and influence clinical decision-making, as illustrated by various examples in this paper. Raising awareness of the impact of race and ethnicity will equip future research leaders with the knowledge to critically evaluate and improve these algorithms. This effort should be supported by equitable structures and systems within research institutions and funding bodies.

## Limitations

The collection and use of racial and ethnic data in healthcare differ widely across countries. This review predominantly focuses on research in the UK and the US, reflecting their sociopolitical histories and the availability of large health datasets with recorded racial and ethnic identities. Countries such as France, Canada, Germany and the Netherlands have limited ethnicity data collection owing to legal and cultural restrictions, which may render some recommendations developed in Anglo-American settings less applicable. Future research must explore how algorithmic bias manifests in diverse healthcare systems and how fairness can be achieved without relying on racial data.

While the discussion primarily addresses race-related biases, the underlying principles and strategies are broadly relevant to other protected characteristics such as gender, disability, and socioeconomic status. However, the focus of the review on race may inadvertently narrow the scope, underemphasizing intersectional biases and the interplay of multiple identities, which can interact and compound inequities in algorithmic outcomes. Intersectionality theory provides a framework for understanding how overlapping identities, such as race, gender, class, and disability, interact to produce compounded forms of disadvantage.^102^ For example, a clinical algorithm may misclassify Black women not only due to racial bias but also because of gendered assumptions embedded in the data and design.

While this review provides an overview of technical solutions, such as fairness metrics or statistical debiasing, these can also obscure the need for structural change. These tools are valuable, but they must be situated within a broader ethical and political framework that acknowledges the historical roots of racial bias in medicine and the ongoing impact of systemic racism.

## Future Priorities for Epidemiologists

We have focused on the development and reporting of new clinical algorithms, however, many will never be implemented in a clinical setting.^83^,^103^ There are several reasons for low implementation, including a lack of transparency and limited IT infrastructure. In their comprehensive review, The AHRQ expressed concern over the lack of real-world evidence where disparities are potentially exacerbated due to structural inequities.^2^ For example, a recent review found that no clinical algorithms developed using EHRs and AI have been appraised in a real-world setting.^83^ Therefore, instead of developing new algorithms, research efforts could refocus on observing how implemented algorithms play out in clinical settings in terms of effectiveness, cost, and equity. Regular review and updating of existing algorithms to reflect evolving data and societal contexts should also be prioritized. Otherwise, the promise of equitable healthcare risks is undermined by the continued perpetuation of historical bias.

## Conclusion

Dismantling racial bias in clinical algorithms presents a complex challenge that demands sustained attention from development through implementation. Researchers and clinicians have begun replacing race-based adjustments with race-neutral algorithms in areas such as pulmonary function testing and obstetrics, demonstrating that fairness can be improved without compromising diagnostic accuracy. However, the outcomes of these changes vary across clinical contexts. In some cases, it remains unclear whether race-neutral approaches reduce or inadvertently reinforce disparities highlighting the need for rigorous evaluation and proactive strategies to identify and mitigate bias.

Future efforts by epidemiologists and health data scientists should focus on incorporating diverse data sources, capturing the true social and biological determinants of health, implementing bias detection and fairness mitigation strategies, ensuring transparent reporting, and engaging with diverse communities to understand specific healthcare needs. Additionally, educating students and trainees on race as a sociopolitical construct is an important step towards raising awareness and achieving health equity. It is also essential to monitor, evaluate, and refine our approaches in real-world settings to ensure that clinical algorithms serve all patients equally and effectively.

At the same time, algorithmic bias must be recognized not merely as a technical flaw, but as a reflection of deeper structural inequities embedded in the data and systems that epidemiologists engage with. Addressing this bias requires more than statistical fixes - it demands a commitment to justice, transparency, and accountability in how data are collected, interpreted, and applied. For epidemiologists, algorithmic equity is not just a theoretical concern but a professional and ethical responsibility. By critically examining the assumptions behind data and models, advocating for inclusive practices, and collaborating across disciplines, epidemiologists can play a pivotal role in ensuring that algorithms promote health equity. This is not only a methodological challenge but a moral imperative.
